# National income inequality and suicide mortality across 170 countries, 2000–2021: within-country evidence and sensitivity to exposure uncertainty, with implications for health communication

**DOI:** 10.3389/fpubh.2026.1915415

**Published:** 2026-09-08

**Authors:** Guoliang Lu, Xinyun Tang

**Affiliations:** 1 School of Journalism and Communication, Chongqing University, Chongqing, China; 2 Institute of Ecological Sociology, Fujian Academy of Social Sciences, Fuzhou, China; 3 School of Journalism and Communication, Shanghai International Studies University, Shanghai, China

**Keywords:** ecological study, fixed effects, income inequality, multiple imputation, public mental health, social determinants of health, suicide mortality

## Abstract

**Introduction:**

Income inequality is a distal population exposure, and ecological panel data cannot identify individual-level suicide mechanisms. We examined whether within-country changes in national disposable-income inequality were associated with changes in suicide mortality and assessed how the estimated association depended on exposure uncertainty, outcome measurement, sample composition, population weighting, and temporal identifying assumptions.

**Methods:**

We linked World Health Organization (WHO) age-standardized suicide rates with Standardized World Income Inequality Database (SWIID) disposable-income Gini estimates for 170 countries from 2000 to 2021 (3,257 country-years). The primary specification was a fully adjusted, unweighted two-way fixed-effects model with country-clustered standard errors. All 100 completed SWIID version 9.92 series were pooled using Rubin’s rules and Barnard–Rubin degrees of freedom. Sensitivity analyses examined population weighting, temporal specifications, alternative outcomes, reporting and measurement-quality subsets, and country influence. We also conducted an observed-panel simulation and equivalence testing within ±0.010 log-rate units per Gini point.

**Results:**

The posterior-mean plug-in estimate was b = −0.0157 (95% CI, −0.0286 to −0.0027; *p* = 0.018), whereas the SWIID multiple-imputation estimate was b = −0.00831 (95% CI, −0.01688 to 0.00026; *p* = 0.057; fraction of missing information = 0.173). This sensitivity bracket was not treated as formal bounds and straddled the post hoc smallest effect size of interest of |b| = 0.010. Population- and log-population-weighted estimates were −0.00861 (95% CI, −0.02166 to 0.00443; *p* = 0.193) and −0.00864 (95% CI, −0.01718 to −0.00010; *p* = 0.047), respectively. Country-trend, region-year, first-difference, and dynamic estimates were smaller and less precise. All 100 completed-data coefficients and all 170 leave-one-country-out posterior-mean coefficients were negative, while subsets with narrower WHO uncertainty intervals.

## 1 Introduction

Suicide is a major public-health outcome resulting from the interplay of individual vulnerability with social, economic, institutional, and cultural conditions. Clinical disorders, substance use, exposure to violence, social isolation, access to lethal means, health-care availability, and crisis-response systems all contribute to risk. At the population level, economic shocks, labor-market disruption, welfare protection, migration, social cohesion, and mortality-registration practices can also shift observed suicide rates. This multilevel etiology makes suicide relevant to research on the social determinants of mental health, but it also makes ecological associations particularly difficult to interpret. In the present study, both exposure and outcome are measured at the country-year level; the research question is therefore whether national changes in income inequality are associated with national changes in suicide mortality, not whether more unequal individuals face greater suicide risk or whether any individual-level psychosocial mechanism mediates that association. A national indicator can be associated with suicide because it changes the population context, tracks other macro-social changes, is measured differently across countries, or because the statistical model compares fundamentally different sources of variation.

Income inequality is one such national indicator. It describes the distribution of economic resources and may shape population contexts through residential and educational segregation, political influence, access to services, labor-market insecurity, and social protection. Theories of relative deprivation, status anxiety, social comparison, and social cohesion offer possible contextual explanations for why national inequality and population mental health might co-vary (1–7). These are not mechanisms tested here: a country-level Gini does not measure individuals’ perceived rank, anxiety, comparison processes, or social ties, and national suicide mortality does not identify the individuals exposed to those experiences.

The empirical record remains inconsistent. Reviews of income inequality and mortality, depression, subjective well-being, and other mental-health outcomes report positive, null, and occasionally reversed associations, with results varying by geographic scale, outcome, inequality measure, model specification, and study period (4–6, 8). Earlier country- and subnational-level studies also differ (9–12), and three recent cross-national reports have examined Gini coefficients or economic correlates in relation to suicide mortality using other panels, inequality definitions, and estimands (13–15). Accordingly, the novelty of the present study does not lie in establishing a new direction for the ecological inequality–suicide association. Its principal contribution is methodological: it demonstrates how the magnitude and interpretation of that association vary across pooled, between-country, and within-country estimands; alternative treatments of SWIID exposure uncertainty; population weights; outcome series and reporting subsets; sample composition; and temporal identifying assumptions.

Objective and perceived inequality are not interchangeable. A Gini coefficient summarizes an income distribution; it does not reveal whether residents notice it, whom they compare themselves to, whether they regard disparities as legitimate, or whether they expect future mobility. Perceived inequality has been linked to status anxiety and lower social trust, which in turn predict poorer subjective well-being (16). However, the psychological appraisal of the same objective distribution can vary across places and periods (17). Communication environments may further shape how objective inequality becomes publicly visible and interpreted. Digital platforms and online news can circulate narratives of mobility, competition, exclusion, or unfairness; however, these remain possible contextual processes rather than mechanisms observed in the present data. The tunnel hypothesis offers one possible contextual interpretation: visible gains among others may temporarily sustain optimism about one’s own prospects during economic transformation (18), whereas mobility beliefs and perceived competitiveness may modify how inequality is experienced (19, 20). Because this study does not observe any of these individual perceptions or responses, they are used only to interpret possible population contexts and not as tested mediators. A country-level Gini is therefore treated as a distal contextual exposure rather than a direct measure of relative deprivation, social comparison, status anxiety, trust, cohesion, or belonging.

Suicide is also not interchangeable with average distress or life satisfaction. It is a rare mortality event concentrated in vulnerable population segments and shaped by clinical severity, impulsivity, means availability, help-seeking, and institutional response. Durkheim’s account of social integration and regulation provides a historical bridge between suicide and contemporary theories of trust and belonging (38), but national suicide rates are also affected by alcohol environments, age and sex composition, religious and legal norms, medical certification, stigma, and the completeness of death registration. Even age-standardized and modeled estimates cannot eliminate all ascertainment differences. A convincing ecological analysis must therefore distinguish substantive patterns from artifacts of outcome series and reporting quality.

A central statistical challenge is distinguishing between-country and within-country associations. A pooled coefficient is dominated by persistent differences among countries. Countries with high average inequality also differ from those with low average inequality in institutions, welfare regimes, economic structure, culture, religion, population composition, and statistical capacity. These durable differences can confound a cross-sectional association even when annual indicators are included. A within-country coefficient instead asks whether years when a country is more unequal than its usual level are also years when its suicide rate differs from its usual level, after accounting for common time effects and measured covariates. Country fixed effects remove time-invariant national characteristics, but they do not remove time-varying confounding, reverse timing, measurement error, or common trajectories (21–23).

The difference matters particularly when outcomes vary primarily across countries and exposures change slowly within countries. Under those conditions, the fixed-effects coefficient is identified by a small residual component of the exposure. Slow declines or increases in both variables can create a within-country slope even if short-run changes are unrelated. Country-specific trends, region-by-year fixed effects, first differences, and lagged-outcome models progressively remove low-frequency or persistent variation. None is automatically the correct causal model: trends may absorb meaningful gradual pathways, first differences can amplify measurement error, and lagged dependent variables in finite fixed-effects panels can be biased (24). Their value is diagnostic. If the coefficient changes substantially, the conclusion depends on which temporal variation is treated as informative.

Measurement uncertainty creates a related problem. Harmonized inequality databases improve comparability across heterogeneous surveys and definitions, but annual estimates include modeled or imputed components. A posterior mean is smoother than many individual completed series and can appear more precise when treated as observed. SWIID provides 100 completed series to represent uncertainty in the inequality archive (25). Rubin pooling summarizes results across those series, but its frequentist interpretation depends on compatibility between the imputation and substantive models (26, 27). Because SWIID was not constructed using suicide or the present outcome model, neither the posterior-mean plug-in nor the pooled estimate is automatically closer to the latent true Gini association.

The present study asks how the estimated country-level income-inequality–suicide association, and the claims that can reasonably be drawn from it, change with analytical choices in a global country-year panel. Its contribution is methodological rather than a claim to establish the direction of the association or an individual mechanism. First, it reports pooled, between-country, and within-country estimands in the same framework. Second, it propagates all 100 SWIID completed series without mislabeling this step as measurement-error correction. Third, it compares unweighted, population-weighted, and log-population-weighted TWFE estimands. Fourth, it decomposes the mortality-reporting result by region, income, outcome precision, SWIID precision, and within-country Gini variation and treats measurement quality and selection as competing readings. Fifth, it adds an observed-panel estimator-recovery simulation and a smallest-effect-size/equivalence assessment. Throughout, the unit of analysis is the country-year, and the inference is ecological: the design evaluates a population-level association. It cannot test individual-level mediation or the effect of a specific policy intervention.

## 2 Materials and methods

### 2.1 Study design and reporting

We conducted an ecological longitudinal analysis of country-year observations from 2000 through 2021. The unit of analysis was the country-year, and the primary estimand concerned temporal deviations within countries rather than individual suicide risk. Reporting was guided by STROBE (39). No preregistration or dated prospective analysis plan was included with the assembled archive; we therefore do not claim prospective prespecification. At revision, we designated the fully adjusted unweighted TWFE model as the primary analytic model. Population weights, reporting decompositions, alcohol adjustment, equivalence tests, and simulation were reviewer-requested sensitivity or exploratory analyses.

The primary model was unweighted, giving each observed country-year equal influence and targeting a typical country-year in the analytic panel. We also estimated population-weighted and log-population-weighted TWFE models using World Development Indicators total population. Population weighting shifts the estimand toward person-weighted global experience and gives highly populous countries dominant leverage; log-population weighting is an intermediate estimand. All three weighting schemes were applied across the 100 SWIID-completed series. None resolves the ecological limitation.

### 2.2 Primary outcome

The primary outcome was WHO Global Health Observatory indicator MH_12, the age-standardized suicide mortality rate per 100,000 population for both sexes (28). WHO Global Health Estimates (GHE) 2021 directly used cause-of-death registration meeting its inclusion criteria for 66 countries or territories and used GBD 2021 and other modeled inputs where usable registration was unavailable (29, 30). All GHE outputs remain estimates after redistribution, interpolation/extrapolation, and scaling. We retained positive 2000–2021 point estimates and log-transformed the rate; a coefficient b corresponds to 100 × [exp(b) − 1] percent difference per one Gini point.

The public MH_12 extract contains point estimates and lower and upper uncertainty limits but no joint posterior draws and no country-year source flag distinguishing direct registration from modeled input. An exact count of the 3,257 analytic rows by those source classes therefore cannot be reconstructed without nonpublic metadata. We report the WHO framework-level count of 66 directly usable registration systems and use exact overlap with the WHO Mortality Database (1,739 rows in 101 countries) as a reproducible reporting-subset proxy, not as a claim that every overlapping MH_12 value is registration-based. Outcome-interval strata and inverse-variance weighting are diagnostics, not corrections.

### 2.3 Income-inequality exposure

The primary exposure was the Gini coefficient of equivalized disposable household income from SWIID version 9.92, expressed on the 0–100 scale (25). SWIID standardizes inequality observations toward Luxembourg Income Study-compatible concepts using inequality source data, welfare definition, equivalence scale, source relationships, and information from proximate years within countries. Its public construction does not condition on suicide mortality or on the exact four-covariate substantive model used here. Descriptive and plug-in models used the posterior mean; uncertainty analyses used all 100 completed disposable-income Gini series. This archive is therefore not congenial with the suicide analysis in the strict Rubin–Meng sense (26, 27).

Two alternative distributional measures were used. The first was the SWIID market-income Gini, which describes inequality before taxes and transfers. The second was the top-10% share of pretax national income among equal-split adults from the World Inequality Database (31). The top-share coefficient was reported per 10-percentage-point increase to facilitate interpretation. We also examined one- and two-year lags of disposable-income inequality and a quadratic specification to assess delayed or nonlinear patterns.

### 2.4 Covariates and their causal status

Four annual covariates were obtained from the World Bank World Development Indicators (32): gross domestic product per capita at purchasing-power parity in constant 2021 international dollars, unemployment as a percentage of the labor force, urban population as a percentage of the total, and individuals using the internet as a percentage of the population. GDP per capita was log-transformed. The remaining covariates were retained on their percentage-point scales. Internet use was retained as a country-level indicator of digital connectivity and broader structural change, rather than as a measure of individual media exposure, platform use, or communication behavior.

These covariates can co-vary with both income distribution and suicide, but their causal roles are not unique. In a simplified causal graph, prior and concurrent macroeconomic structure can cause both inequality and suicide; GDP, unemployment, urbanization, and internet diffusion may partly measure that structure, whereas some may also lie on pathways from policy or economic change to inequality and suicide (33). The adjusted coefficient is therefore defined descriptively: the conditional within-country association between annual Gini deviations and logged suicide after removing country means, global year shocks, and linear adjustment for the four observed annual covariates. It is not a controlled direct or total causal association. Alcohol policy and consumption, lethal-means restriction, austerity, social expenditure, labor-market restructuring, migration, conflict, and death-certification changes remain plausible time-varying common causes.

### 2.5 Exploratory contextual variable and validation outcomes

We used World Bank income-group classifications for heterogeneity analysis. We also used WHO regions to define region-by-year fixed effects and leave-one-region-out diagnostics. We retained Hofstede individualism scores (34) only for exploratory contextual analysis. Individualism is time-invariant, predates much of the panel, and is available for a restricted set of countries; it cannot serve as a direct measure of the hypothesized individual-level mediators.

**Figure 1:**
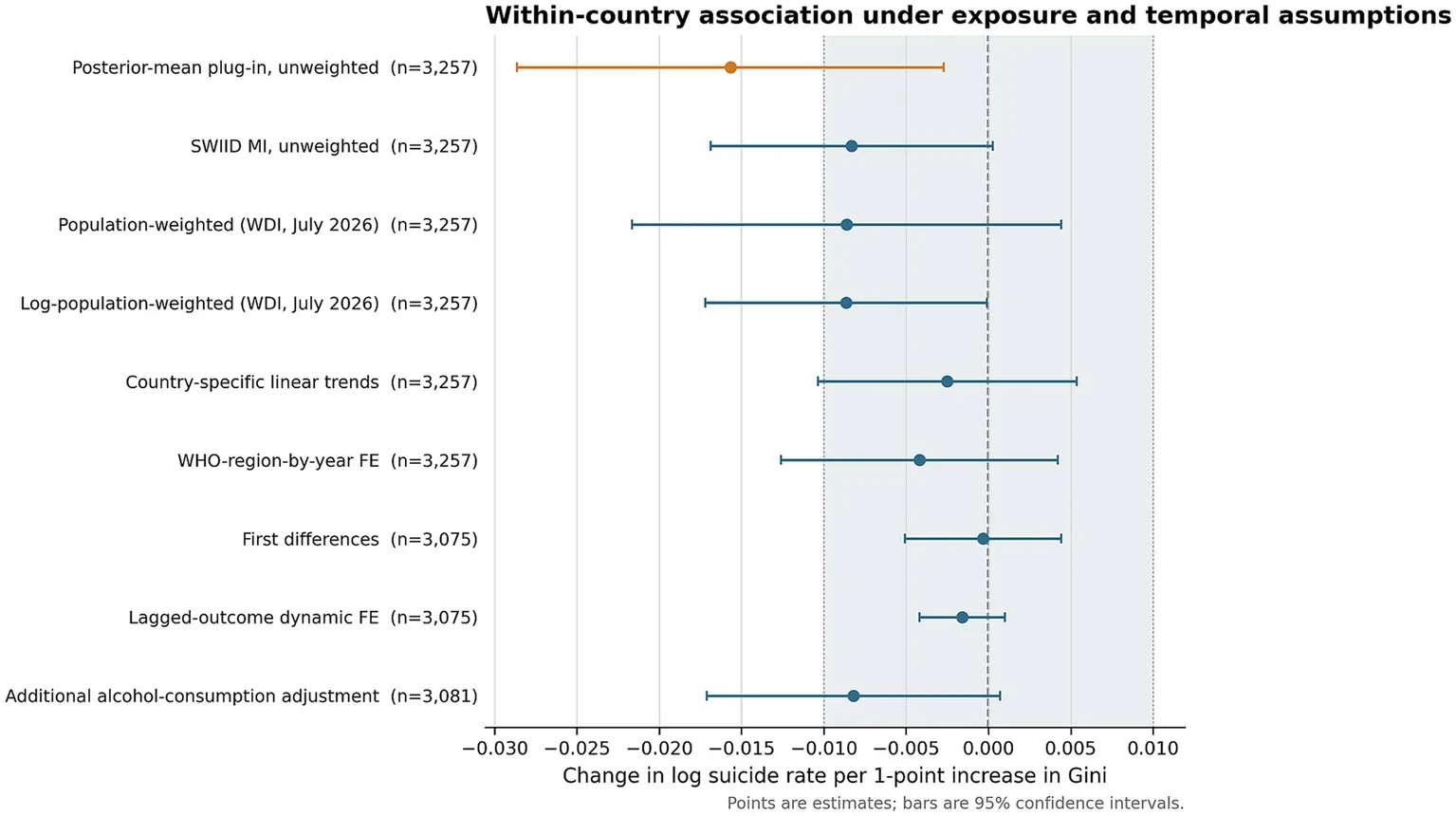
Primary, population-weighted, temporal-identification, alcohol, and outcome-uncertainty estimates. Points represent coefficients, and bars indicate 95% confidence intervals. The shaded band marks the *post hoc* SESOI of ±0.010 log-rate units per Gini point. The posterior-mean plug-in row is shown for comparison; other rows use SWIID multiple imputation.

We evaluated robustness to the suicide measure with GBD 2021 self-harm mortality and WHO Mortality Database reported mortality. GBD models suicide with CODEm using vital registration, verbal autopsy, surveillance, spatial–temporal borrowing, and covariate selection. The GBD 2021 suicide candidate covariates include alcohol consumption per capita, major depressive disorder, non-partner sexual violence, religion, temperature, health-care access and quality, population-density categories, education, lag-distributed income per capita, and the Socio-demographic Index; the published list does not include Gini or SWIID inequality (30, 35). Thus, modeled outcomes may partially encode development and alcohol information. The Mortality Database analysis uses reported deaths but remains vulnerable to underregistration and misclassification (Figure 1).

### 2.6 Country harmonization and analytic sample

Country names were harmonized to ISO 3166-1 alpha-3 codes. The raw WHO both-sex 2000–2021 extract contained 4,312 country-years in 196 countries. Ten zero values (eight for Antigua and Barbuda and two for Saint Vincent and the Grenadines) were excluded before logarithmic transformation. Sequential complete-case counts were 4,302 after the positive-outcome filter, 3,432 after SWIID, 3,374 after GDP, 3,305 after unemployment, 3,305 after urbanization, and 3,259 after internet use. Bahrain and Somalia then had only one complete row each and were excluded, leaving 3,257 rows in 170 countries. No country-year duplicates were present, and no primary variable was linearly interpolated.

**Figure 2:**
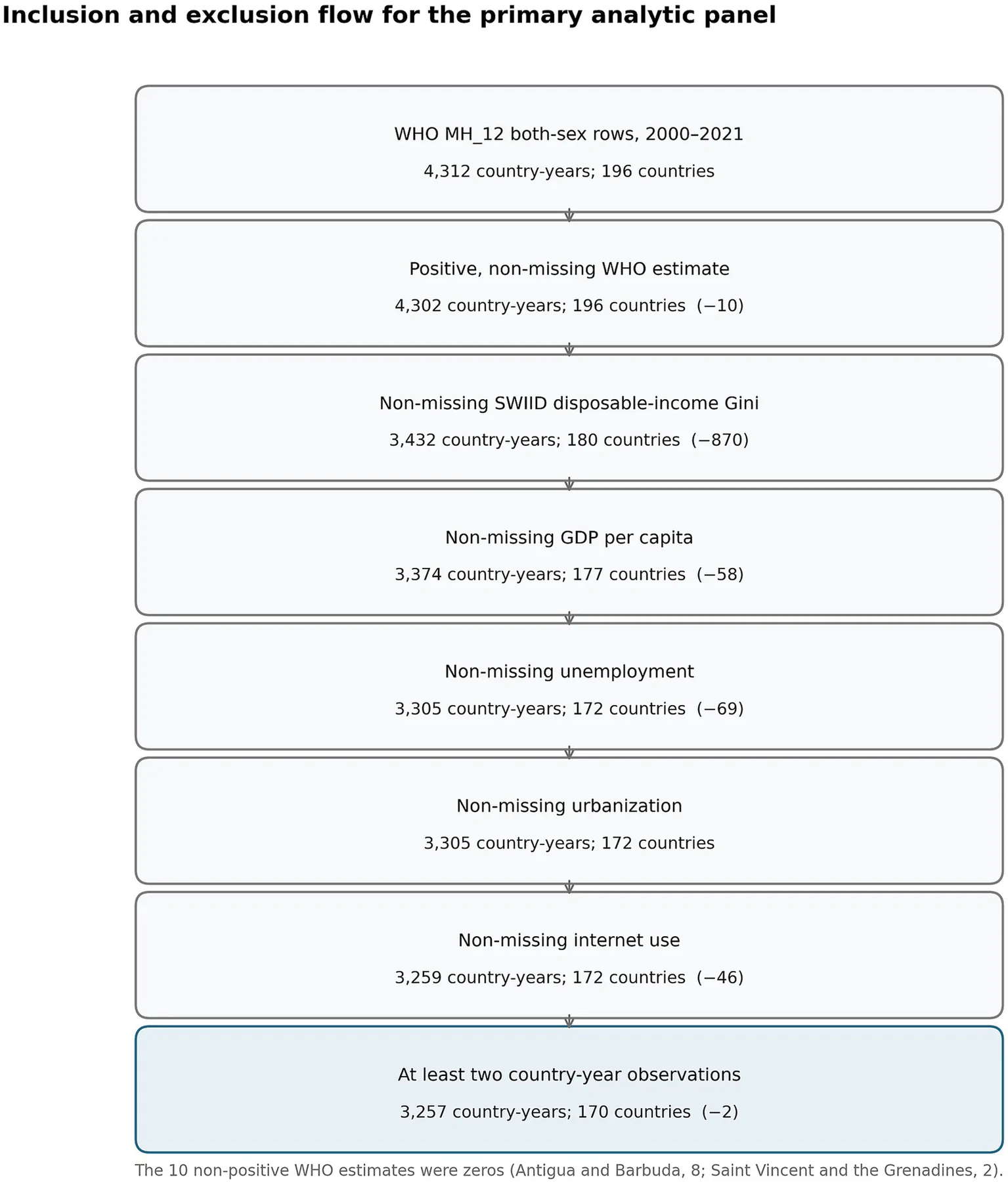
Inclusion and exclusion flow from the raw WHO both-sex country-year extract to the 170-country analytic panel.

A balanced 170-country panel over 22 years would contain 3,740 observations; coverage was 87.1%. Countries contributed a mean of 19.2 years, a median of 22, and a range of 5–22; 93 countries were complete, and 113 contributed at least 20 years. For first differences and the dynamic model, the reduction from 3,257 to 3,075 observations comprises the first observation in each of 170 countries plus 12 observations following internal calendar gaps; no further restriction was applied (Figure 2).

### 2.7 Descriptive decomposition and scale

We calculated overall and within-country standard deviations for the outcome, exposure, and covariates. The within-country component of a variable was defined as its annual value minus its country-specific observed-period mean. For logged suicide, we also calculated the share of observation-level variance attributable to country means. This descriptive quantity shows how strongly a pooled analysis is dominated by persistent country differences; it is not presented as a random-effects intraclass correlation.

For conventional fixed-effects models, the disposable-income Gini was centered on the analytic-sample mean. For the within-between model, we decomposed each time-varying predictor into two terms: the annual deviation from the country mean, and the country mean centered on the grand mean. The former estimates a within-country association, whereas the latter estimates a between-country association conditional on the corresponding covariate decomposition.

### 2.8 Primary statistical models

We began with pooled ordinary least squares including year fixed effects and the four covariates. This model describes the combined cross-sectional and temporal association and is expected to be dominated by between-country variation. We next estimated a within-between, or Mundlak, model with year fixed effects and country-clustered standard errors. Conceptually, the outcome was regressed on each predictor’s within-country deviation and the centered country mean. Including both components for the Gini and all covariates prevents the within coefficient from being conflated with cross-country differences in covariate means.

The designated primary specification was an unweighted TWFE model with country and year indicators, log GDP per capita, unemployment, urbanization, and internet use. Country indicators remove factors constant within a country over the observed period; year indicators remove shocks common to all countries. The coefficient is identified by deviations from each country’s average inequality after accounting for the observed covariates and common annual shifts. Country-clustered standard errors allow arbitrary serial correlation and heteroskedasticity within countries. Two-way country-and-year clustered inference, implemented using multiway cluster-robust covariance estimation (36), was supplementary because only 22-year clusters were available.

Adjustment was introduced in stages: unadjusted country and year fixed effects; addition of log GDP per capita; unemployment; urbanization; and internet use. The fully adjusted unweighted TWFE coefficient is the primary conditional association, not a causal quantity. Population weighting, temporal restrictions, alternative outcomes, precision strata, and other models target different or selected estimands.

### 2.9 Sensitivity to temporal identification

Four specifications examined whether the reference result depended on low-frequency co-movement. First, country-specific linear time trends allowed each country to follow its own gradual trajectory. Second, WHO-region-by-year fixed effects replaced common year effects while country fixed effects were retained, absorbing annual shocks shared within geographic regions. Third, first-difference models related year-to-year change in logged suicide to year-to-year change in inequality and the covariates. Fourth, a dynamic fixed-effects model included the previous year’s logged suicide rate.

These models do not form a hierarchy in which the most saturated specification is necessarily correct. Country trends can remove gradual causal pathways together with confounding trajectories. Region-year effects can absorb policy or economic changes that are substantively part of the exposure process. First differences emphasize short-run changes and can worsen attenuation from measurement error. Dynamic fixed-effects estimation can be biased when the number of periods is finite (24). We therefore used attenuation or sign changes across these specifications as evidence that the association is sensitive to temporal identifying assumptions, not as a mechanical test that one model is true and the others are false.

### 2.10 Propagation of SWIID uncertainty

SWIID version 9.92 provides 100 completed series that capture uncertainty arising from source comparability, missing distributional information, and the harmonization model. We fitted each regression to every completed series and combined coefficients and variances using Rubin’s rules (40). Confidence intervals and *p*-values used Barnard–Rubin small-sample degrees of freedom, with countries minus one as the complete-data reference (37). We report the pooled coefficient, total standard error, confidence interval, *p*-value, fraction of missing information (FMI), between-series dispersion, and range. Because the external SWIID model omits this suicide outcome and the substantive model, pooled MI inference is potentially uncongenial (26, 27); it propagates the archive’s uncertainty but is not a classical measurement-error correction and does not recover a latent true Gini.

To examine the direction and possible magnitude of estimator behavior under that uncongeniality, we ran an observed-panel calibration simulation. After residualizing the posterior mean, all 100 completed Gini series, and logged suicide on country and year fixed effects and the four covariates, we imposed a known within-country coefficient of −0.010 and added empirical residuals with country-level Rademacher wild-bootstrap signs. We used 400 replicates under each of two exposure-truth scenarios: the posterior mean as latent exposure and a randomly selected completed series as latent exposure. Each replicate compared the posterior-mean plug-in estimator with Rubin pooling. This simulation is calibrated to the observed panel and is a diagnostic, not a validation of either latent-exposure assumption.

### 2.11 Robustness, influence, and heterogeneity analyses

Robustness analyses included alternative exposure timing and definition, population and log-population weights, inverse-outcome-interval weights, balanced and pre-2020 panels, and alcohol consumption. The reporting result was decomposed using (i) exact Mortality Database-overlap country-years, (ii) all years from countries that ever overlapped, (iii) countries with no overlap, (iv) narrower versus wider country-average WHO uncertainty, (v) smaller versus larger country-average SWIID standard errors, (vi) higher versus lower within-country Gini variation, (vii) period, region, and income groups, and (viii) leave-one-region-out models. To reduce compositional differences, we also compared narrower versus wider country-average WHO intervals within the exact Mortality Database-overlap sample, splitting the 101 overlapping countries at the median of their mean relative interval width. The alternative overlap-versus-non-overlap comparison restricted to high-income rows was audited but not estimated because the high-income non-overlap cell contained only 72 country-years in 18 countries (median 3 years per country; only five countries contributed at least 5 years). These selected comparisons can reflect both reduced measurement error and compositional selection.

We assessed influence by re-estimating the fully adjusted model after deleting each country in turn. We also excluded one WHO region at a time. For income-group heterogeneity, we estimated a single fixed-effects model with Gini-by-income-group interactions and used a joint Wald test of equal within-country slopes. This approach is preferable to interpreting significance within separately estimated strata as evidence of differences between strata.

For sex heterogeneity, male and female WHO series were stacked. The formal comparison included country-by-sex and year-by-sex fixed effects; the Gini and all four macroeconomic covariates were interacted with a male indicator. This parameterization reproduces the fully adjusted female and male two-way fixed-effects slopes within one model and directly tests their difference. Standard errors were clustered by country. We estimated the comparison at the posterior mean and in all 100 SWIID-completed datasets. Income-group and individualism analyses remained posterior-mean exploratory analyses because they addressed secondary questions or had restricted coverage.

### 2.12 Statistical inference and reproducibility

All tests were two-sided. We selected an absolute log-rate coefficient of 0.010 per Gini point—approximately a 1.005% rate difference—as a transparent, *post hoc* smallest effect size of interest (SESOI), chosen for interpretability rather than preregistration. We reported the exact 80 and 90% minimum detectable coefficients and a two-one-sided equivalence test (TOST) for bounds ±0.010 (41). Secondary specifications were not multiplicity-adjusted and are interpreted as sensitivity patterns rather than independent confirmatory tests. Analysis used Python, pandas, NumPy, SciPy, statsmodels, and explicit fixed-effects routines; an independent dummy-variable statsmodels implementation reproduced the principal weighted and unweighted coefficients and standard errors to numerical precision.

## 3 Results

### 3.1 Panel coverage and identifying variation

The primary panel included 3,257 observations from 170 countries. The mean age-standardized suicide rate was 10.29 per 100,000 population, and the mean disposable-income Gini was 38.80 (Table 1). The overall standard deviation of the Gini was 8.14 points, but its within-country standard deviation was only 1.44 points. Logged suicide had an overall standard deviation of 0.767 and a within-country standard deviation of 0.164. Approximately 95.4% of the observed variance in logged suicide was attributable to differences in country means. Consequently, a pooled cross-national regression primarily contrasts countries with persistently different suicide levels rather than isolating annual national change.

**Table 1:** Descriptive statistics for the primary analytic panel (170 countries, 3,257 country-years, 2000–2021).

Variable	Mean	Total SD	Within SD	Min	Max	*N*
Suicide rate (per 100,000)	10.29	6.71	2.13	0.61	47.88	3,257
Log suicide rate	2.090	0.767	0.164	−0.491	3.869	3,257
Gini, disposable income	38.80	8.14	1.44	22.30	65.70	3,257
Gini, market income	45.63	6.41	1.43	31.30	72.40	3,257
GDP per capita (PPP, 2021$)	21,620	23,073	3,874	712	145,591	3,257
Unemployment (%)	7.90	5.82	2.14	0.14	37.32	3,257
Urban population (%)	57.15	22.10	3.15	8.04	100.00	3,257
Internet users (%)	34.70	30.92	18.59	0.00	99.50	3,257

Within SD is the standard deviation of annual deviations from each country’s observed-period mean. GDP per capita is measured at purchasing-power parity in constant 2021 international dollars.

**Figure 3:**
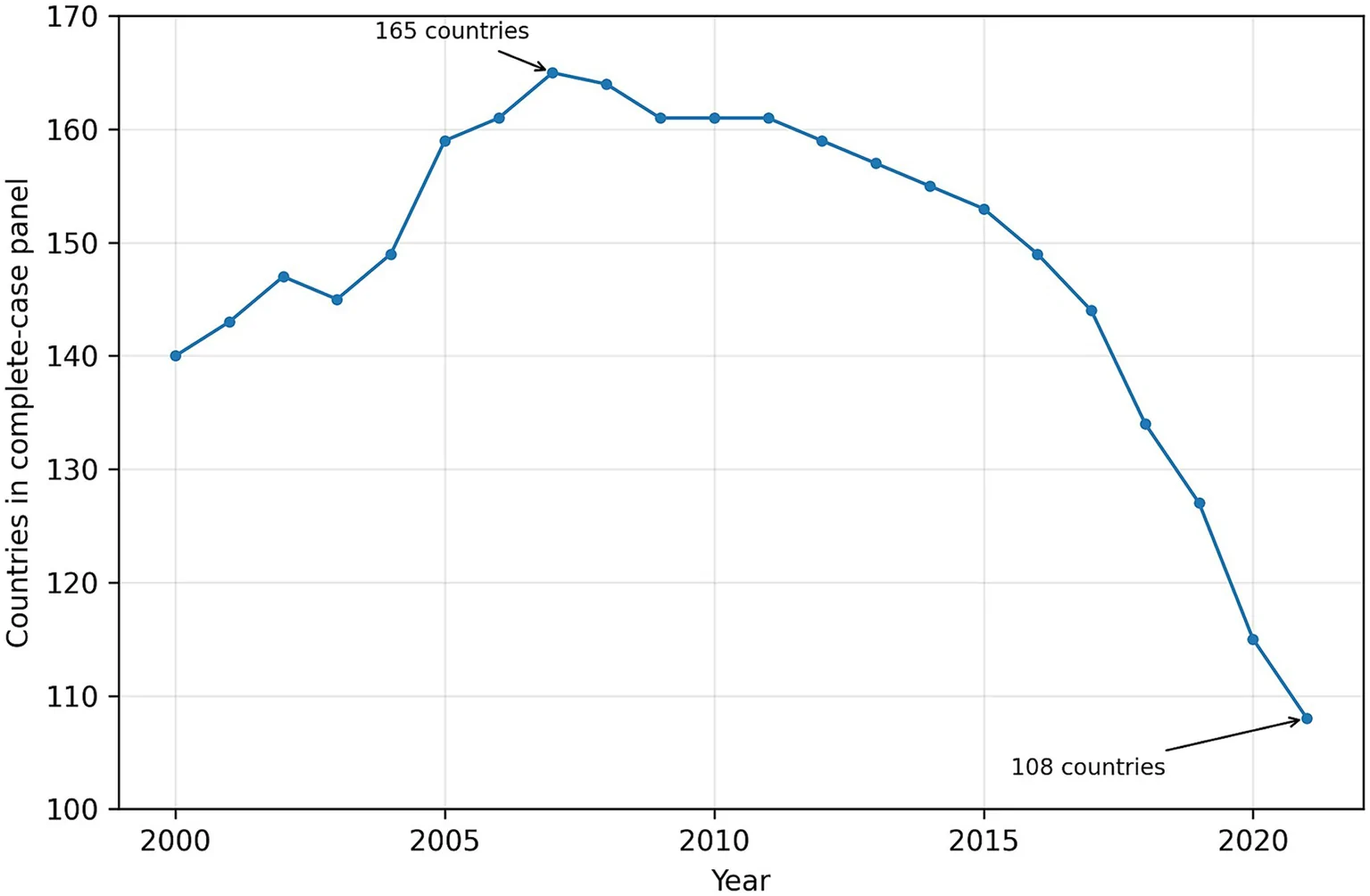
Annual country coverage of the primary analytic panel from 2000 to 2021. Bars show the number of countries included in each year; coverage peaked at 165 countries in 2007 and declined to 108 in 2021.

The panel was broad but unbalanced. Annual coverage peaked at 165 countries in 2007 and declined to 108 countries in 2021 (Figure 3). Ninety-three countries contributed every year, whereas the shortest series contributed five observations. The high proportion of nearly complete national series reduces, but does not eliminate, concern that changing composition drives the findings.

### 3.2 Pooled, between-country, and within-country estimates

Using the SWIID posterior mean, the adjusted pooled association was b = 0.0033 (95% CI, −0.0145 to 0.0211; *p* = 0.715). The hybrid model separated a positive but imprecise between-country coefficient (b = 0.0115; *p* = 0.273) from a negative within-country coefficient (b = −0.0157; 95% CI, −0.0286 to −0.0029; *p* = 0.017). The corresponding MI estimates were b = 0.0043 for the pooled association (*p* = 0.619), b = 0.0126 for the between-country association (*p* = 0.222), and b = −0.00832 for the within-country association (95% CI, −0.01680 to 0.00017; *p* = 0.055). Estimand choice changed both sign and interpretation; the within-country point estimates remained negative.

In the staged TWFE models, the unadjusted coefficient was −0.0119 (SE = 0.0069, *p* = 0.085). Adding log GDP per capita produced little change. Adding unemployment increased the negative magnitude to −0.0142, and further adjustment for urbanization and internet use yielded the fully adjusted coefficient of −0.0157 (SE = 0.0066, *p* = 0.018; Table 2). Two-way clustering by country and year left the point estimate unchanged and produced a similar standard error. Excluding internet use resulted in b = −0.0136 (*p* = 0.043), indicating that the main result did not depend solely on that rapidly changing covariate.

**Table 2:** Income inequality and logged suicide across posterior-mean plug-in, multiple-imputation, and identification-sensitive estimands.

Model/estimand	b	SE	95% CI	*p*	Country-years
Posterior mean: adjusted pooled OLS + year FE	0.0033	0.0090	[−0.014, 0.021]	0.715	3,257
Posterior mean: hybrid between-country Gini	0.0115	0.0105	[−0.009, 0.032]	0.273	3,257
Posterior mean: hybrid within-country Gini	−0.0157	0.0065	[−0.028, −0.003]	0.017	3,257
Posterior mean: fully adjusted TWFE	−0.0157	0.0066	[−0.029, −0.003]	0.018	3,257
SWIID MI: adjusted pooled OLS + year FE	0.0043	0.0087	[−0.013, 0.021]	0.619	3,257
SWIID MI: hybrid between-country Gini	0.0126	0.0103	[−0.008, 0.033]	0.222	3,257
SWIID MI: hybrid within-country Gini	−0.0083	0.0043	[−0.017, 0.000]	0.055	3,257
SWIID MI: fully adjusted TWFE	−0.0083	0.0043	[−0.017, 0.000]	0.057	3,257
SWIID MI: country-specific linear trends	−0.0025	0.0040	[−0.010, 0.005]	0.529	3,257
SWIID MI: country FE + WHO region-year FE	−0.0042	0.0042	[−0.013, 0.004]	0.325	3,257
SWIID MI: first differences	−0.0003	0.0024	[−0.005, 0.004]	0.892	3,075
SWIID MI: dynamic FE + lagged suicide	−0.0016	0.0013	[−0.004, 0.001]	0.224	3,075

MI pools all 100 SWIID version 9.92 completed series. Fully adjusted models include log GDP per capita, unemployment, urbanization, and internet use; standard errors are clustered at the country level. Barnard–Rubin degrees of freedom are used for MI. Temporal models target different identifying variation and are not ranked as successively more correct.

On the log-rate scale, b = −0.0157 corresponds to a 1.6% decrease in suicide for a one-point increase in the Gini. A one-within-country-standard-deviation difference of 1.44 Gini points corresponds to approximately a 2.2% lower suicide, with a confidence interval of roughly −4.0 to −0.4%. This calculation describes the fitted association across the variation observed in the panel. It should not be interpreted as the expected effect of a policy that intentionally increases inequality.

### 3.3 Exposure uncertainty and temporal-identification sensitivity

The primary SWIID MI model yielded b = −0.00831, SE = 0.00433, 95% CI − 0.01688 to 0.00026, *p* = 0.057, and Barnard–Rubin df = 132.7. FMI was 0.173, and the between-completion standard deviation was 0.00179. All 100 completed-data coefficients were negative (range, −0.01296 to −0.00385). The pooled estimate was about 47% smaller than the posterior-mean plug-in estimate, but uniform sign and the uncongenial external imputation model argue against describing the result as absence. We use −0.0157 to −0.0083 as an assumption-dependent sensitivity bracket (Table 3).

**Table 3:** Suicide-outcome cross-validation and formal sex and income-group comparisons before and after SWIID uncertainty propagation.

Analysis	Exposure handling	Estimate/test	SE	*p*	Observations
GBD/IHME age-standardized outcome	Posterior mean	b = −0.0130	0.0051	0.012	3,257 country-years
GBD/IHME age-standardized outcome	SWIID MI	b = −0.0062	0.0035	0.079	3,257 country-years
WHO modeled outcome, reporting subset	Posterior mean	b = −0.0283	0.0087	0.002	1,739 country-years
WHO modeled outcome, reporting subset	SWIID MI	b = −0.0180	0.0070	0.012	1,739 country-years
WHO Mortality Database reported outcome	Posterior mean	b = −0.0370	0.0119	0.003	1,739 country-years
WHO Mortality Database reported outcome	SWIID MI	b = −0.0218	0.0124	0.084	1,739 country-years
Gini × male interaction	Posterior mean	b = −0.0077	0.0048	0.107	6,514 sex-country-years
Gini × male interaction	SWIID MI	b = −0.0039	0.0037	0.294	6,514 sex-country-years
Income-group slope equality	Posterior mean	Wald χ^2^(3) = 4.31	—	0.230	3,257 country-years

Outcome rows are from fully adjusted fixed-effects models with country-clustered standard errors. The Mortality Database overlap is a selected reporting subset, not a row-level MH_12 source classification. The sex model includes country-by-sex and year-by-sex fixed effects as well as interactions among sex and the Gini and all four covariates.

Country-specific trends yielded b = −0.00250 (95% CI, −0.01037 to 0.00536; *p* = 0.529; FMI = 0.338), WHO-region-by-year effects yielded b = −0.00419 (95% CI, −0.01260 to 0.00421; *p* = 0.325; FMI = 0.222), first differences yielded b = −0.00032 (95% CI, −0.00507 to 0.00442; *p* = 0.892; FMI = 0.479), and dynamic adjustment yielded b = −0.00159 (95% CI, −0.00418 to 0.00099; *p* = 0.224; FMI = 0.307). These models show that short-run and trajectory-adjusted associations were small and imprecise, whereas their higher FMI also indicates that increasingly residual variation contains more SWIID completion uncertainty. Neither interpretation alone identifies the latent association.

**Figure 4:**
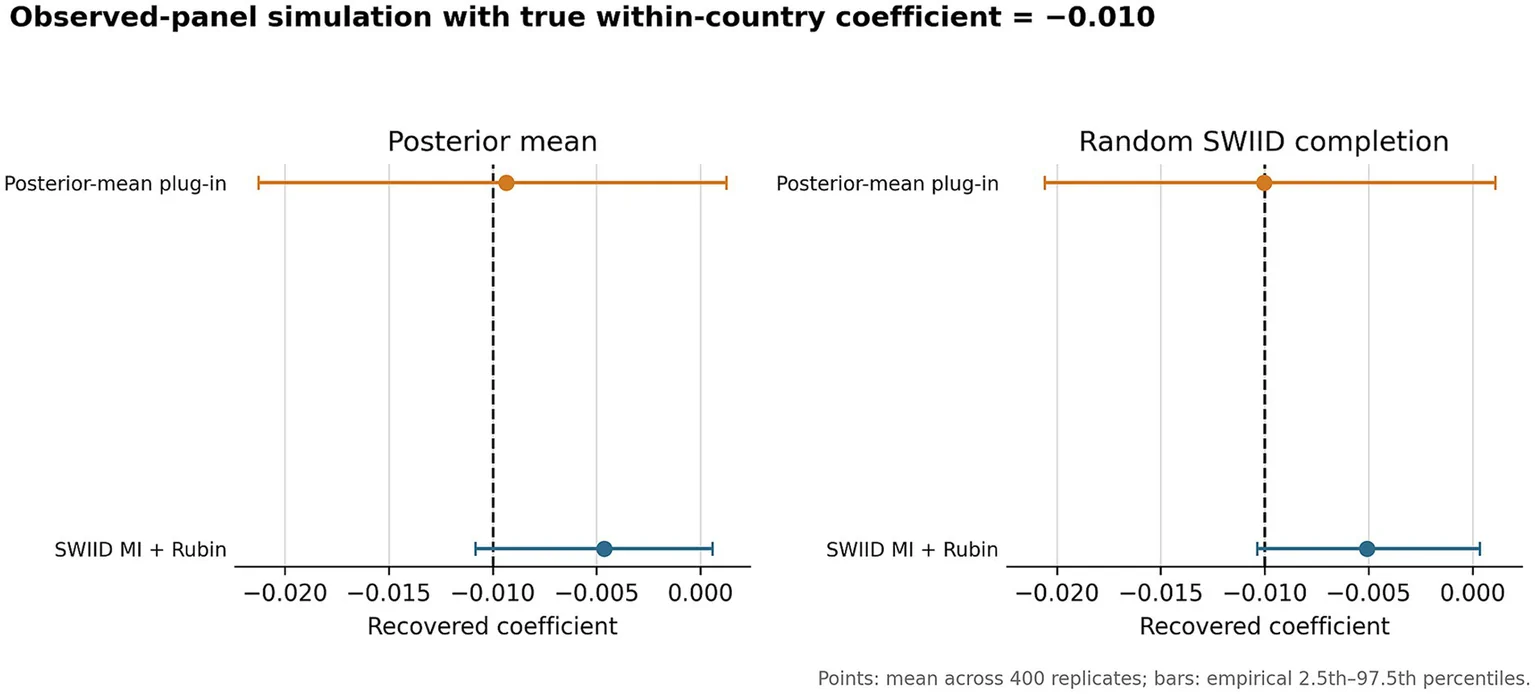
Observed-panel estimator-recovery simulation with a true within-country coefficient of −0.010. Points show the mean estimates across 400 replicates, and bars show the empirical 2.5th–97.5th percentiles under two latent-exposure scenarios. The simulation diagnoses possible attenuation due to uncongeniality but does not identify the true exposure.

In the observed-panel simulation with a true coefficient of −0.010, the posterior-mean plug-in estimator averaged −0.00936 (bias +0.00064; 95% interval coverage 0.978) when the posterior mean was treated as latent and −0.01003 (bias −0.00003; coverage 0.965) when a random completion was latent. Rubin pooling averaged −0.00464 (bias +0.00536; coverage 0.843) and −0.00509 (bias +0.00491; coverage 0.898), respectively. Under these two calibrated data-generating mechanisms, pooling attenuated the imposed slope. The 0.978 plug-in coverage in the posterior-mean-latent scenario is uninformative about exposure-error performance because the generating and fitted exposure are identical in that scenario. Because neither scenario reveals the actual latent Gini, the simulation motivates bracketing rather than declaring the plug-in estimator correct (Figure 4).

### 3.4 Robustness to panel composition, alternative inequality measures, and influence

Population weighting did not reverse the signal. The SWIID MI estimates were −0.00831 unweighted (95% CI, −0.01688 to 0.00026; *p* = 0.057), −0.00861 with population weights (95% CI, −0.02166 to 0.00443; *p* = 0.193), and −0.00864 with log-population weights (95% CI, −0.01718 to −0.00010; *p* = 0.047). Updated July 2026 and locally archived April 2026 population series changed these coefficients by less than 0.000001. With the *post hoc* SESOI ±0.010, the primary MI 90% CI was −0.01549 to −0.00113 and TOST *p* = 0.349, so equivalence was not established. Exact 80 and 90% minimum detectable coefficients were 0.01223 and 0.01415, respectively (Table 4).

**Table 4:** Population weighting, alcohol adjustment, and outcome-precision sensitivity (SWIID multiple imputation).

Specification	b	95% CI	*p*	FMI	*N* (countries)
Unweighted SWIID MI	−0.0083	[−0.0169, 0.0003]	0.057	0.173	3,257 (170)
Population-weighted SWIID MI	−0.0086	[−0.0217, 0.0044]	0.193	0.370	3,257 (170)
Log-population-weighted SWIID MI	−0.0086	[−0.0172, −0.0001]	0.047	0.166	3,257 (170)
Alcohol-available sample, original adjustment	−0.0085	[−0.0174, 0.0004]	0.061	0.184	3,081 (168)
Plus alcohol consumption	−0.0082	[−0.0171, 0.0007]	0.071	0.185	3,081 (168)
Inverse WHO-interval-variance weighted MI	−0.0104	[−0.0302, 0.0094]	0.301	0.175	3,257 (170)

Population values are from World Development Indicators. The alcohol indicator covers 3,081 country-years in 168 countries and is a secondary adjustment because it may be a confounder, mediator, or outcome-model predictor. FMI is the fraction of missing information.

**Figure 5:**
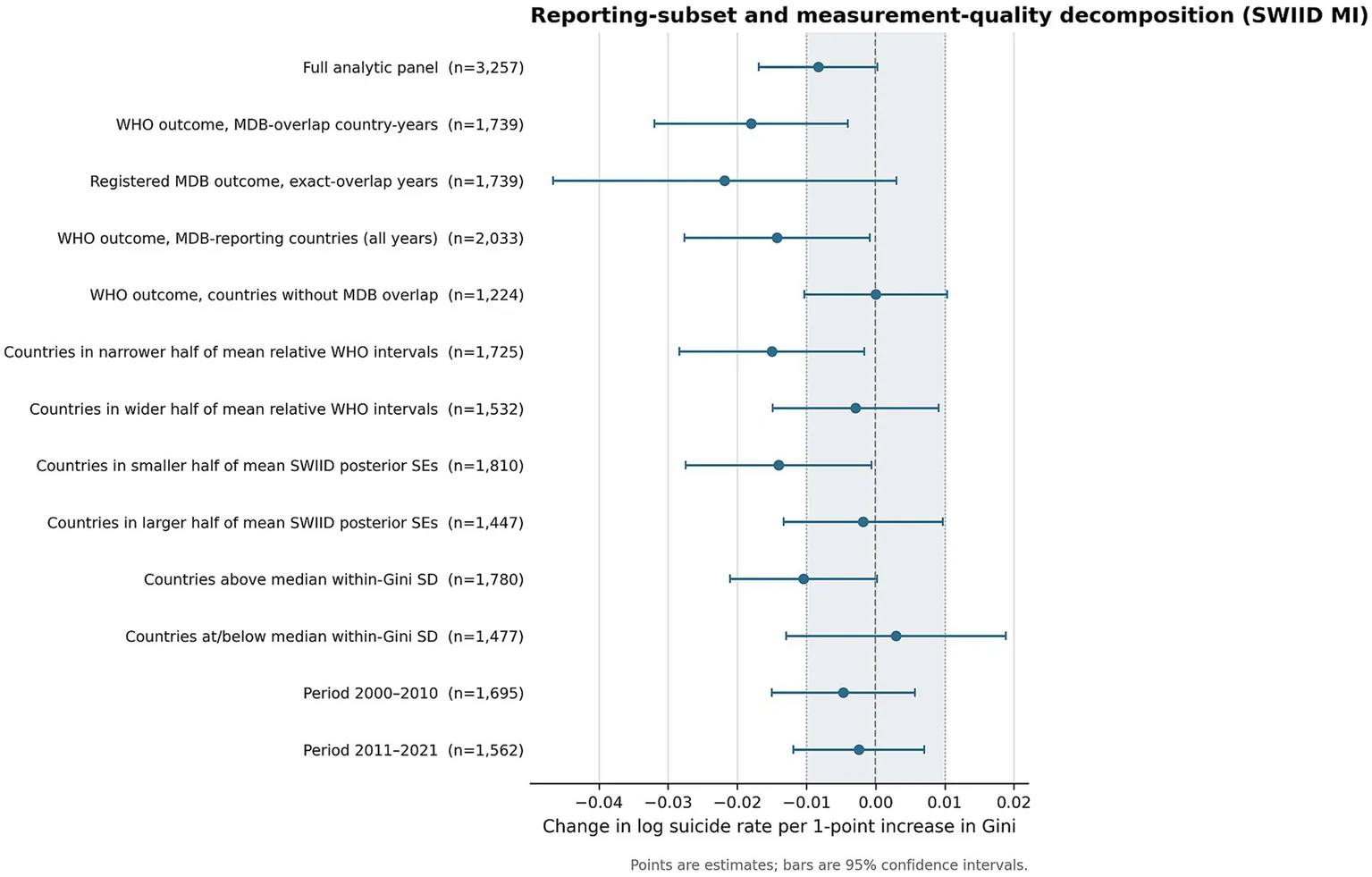
Reporting-subset and measurement-quality decomposition using SWIID multiple imputation. Larger negative coefficients in more precise subsets are compatible with reduced attenuation, compositional selection, or both; the figure does not distinguish among these mechanisms.

The reporting and precision decompositions supported both measurement and selection readings. The exact Mortality Database-overlap rows yielded MI b = −0.01800 (95% CI, −0.03196 to −0.00404; *p* = 0.012), all years in the 101 reporting countries yielded b = −0.01425 (*p* = 0.037), and countries with no overlap yielded b = 0.00001 (*p* = 0.999). The narrower half of country-average WHO intervals yielded b = −0.01500 (*p* = 0.028) versus −0.00290 (*p* = 0.630) in the wider half; the smaller half of country-average SWIID standard errors yielded b = −0.01403 (*p* = 0.041) versus −0.00180 (*p* = 0.754) in the larger half. Within the exact-overlap sample, the comparison yielded b = −0.02325 (95% CI, −0.03859 to −0.00791; *p* = 0.004) for countries with narrower WHO intervals and b = −0.01315 (95% CI, −0.03668 to 0.01038; *p* = 0.265) for those with wider intervals. The high-income overlap/non-overlap comparison was not estimated because its non-overlap cell contained only 72 country-years in 18 countries and only five countries contributed at least five years. However, exact-overlap rows were 58.1% high-income and 54.2% European, compared with 4.7 and 6.9% outside the overlap, and had greater within-country Gini variation. These data cannot separate measurement quality from composition (Table 5; Figure 5).

**Table 5:** Reporting-subset, measurement-quality, and within-stratum decomposition (SWIID multiple imputation).

Selected sample	b	95% CI	*p*	FMI	*N* (countries)
Full analytic panel	−0.0083	[−0.0169, 0.0003]	0.057	0.173	3,257 (170)
Exact Mortality Database overlap	−0.0180	[−0.0320, −0.0040]	0.012	0.217	1,739 (101)
Exact overlap: narrower WHO intervals	−0.0232	[−0.0386, −0.0079]	0.004	0.158	1,057 (51)
Exact overlap: wider WHO intervals	−0.0131	[−0.0367, 0.0104]	0.265	0.210	682 (50)
Reporting countries, all analytic years	−0.0142	[−0.0276, −0.0009]	0.037	0.268	2,033 (101)
Countries with no exact overlap	0.0000	[−0.0103, 0.0103]	0.999	0.320	1,224 (69)
Narrower half of WHO intervals	−0.0150	[−0.0283, −0.0017]	0.028	0.140	1,725 (85)
Wider half of WHO intervals	−0.0029	[−0.0149, 0.0091]	0.630	0.269	1,532 (85)
Smaller half of SWIID standard errors	−0.0140	[−0.0274, −0.0006]	0.041	0.227	1,810 (85)
Larger half of SWIID standard errors	−0.0018	[−0.0133, 0.0097]	0.754	0.397	1,447 (85)
Higher within-country Gini variation	−0.0104	[−0.0210, 0.0002]	0.054	0.109	1,780 (85)
Lower within-country Gini variation	0.0030	[−0.0129, 0.0188]	0.709	0.423	1,477 (85)

Precision and reporting restrictions are selected samples, not randomized quality corrections. Within the exact-overlap sample, countries were split at the median of their mean relative WHO interval width. We did not estimate the high-income overlap/non-overlap contrast because the non-overlap cell was sparse (72 country-years in 18 countries; median 3 years; only five countries had at least 5 years). The exact-overlap sample was more strongly concentrated in high-income countries and Europe and had narrower WHO intervals, smaller SWIID standard errors, and greater within-country Gini variation than rows outside the overlap.

All 170 leave-one-country-out posterior-mean coefficients were negative, statistically distinguishable from zero at *p* < 0.05, and ranged from −0.0183 to −0.0140. Leave-one-region-out MI estimates remained negative, but removal of the Americas (b = −0.00450; *p* = 0.333) or Europe (b = −0.00621; *p* = 0.199) widened intervals. In the 3,081 rows (168 countries) with World Bank alcohol data, the original-adjustment MI estimate was −0.00848 (*p* = 0.061) and the alcohol-adjusted estimate was −0.00821 (95% CI, −0.01711 to 0.00070; *p* = 0.071). Alcohol adjustment did not materially change the point estimate, but it is secondary because alcohol may be a confounder, mediator, or component of modeled suicide outcomes.

### 3.5 Robustness to the suicide outcome

With the GBD 2021 age-standardized self-harm outcome on the same panel, the posterior-mean estimate was b = −0.01297 (95% CI, −0.02300 to −0.00294; *p* = 0.012) and the SWIID MI estimate was b = −0.00622 (95% CI, −0.01316 to 0.00073; *p* = 0.079). The WHO and GBD logged outcomes correlated at 0.94 at the level and 0.70 within countries. Agreement in sign does not constitute independent replication because WHO and GBD share registration inputs and WHO imports GBD 2021 results where usable registration is absent.

Among the 1,739 exact Mortality Database-overlap country-years, the posterior-mean coefficients were −0.02834 for the WHO-modeled outcome and −0.03699 for the registered outcome. With SWIID uncertainty propagated, the WHO outcome yielded b = −0.01800 (*p* = 0.012) and the registered outcome yielded b = −0.02183 (95% CI, −0.04665 to 0.00299; *p* = 0.084; FMI = 0.345). One reading is lower attenuation when both exposure and outcome are better measured. An equally relevant reading is selection on income, Europe, reporting institutions, and identifying variation. The design cannot choose between those explanations, and the overlap is not a row-level source flag for MH_12.

### 3.6 Heterogeneity and exploratory contextual analysis

In the fully interacted stacked model, the posterior-mean female slope was −0.01054 (*p* = 0.127), and the male slope was −0.01826 (*p* = 0.008). Their formal difference was imprecise (Gini-by-male interaction b = −0.00771, SE = 0.00475, *p* = 0.107). After SWIID uncertainty was propagated, the female slope was −0.00575 (*p* = 0.242), the male slope was −0.00966 (*p* = 0.033), and the interaction remained imprecise (b = −0.00391; 95% CI, −0.01125 to 0.00343; *p* = 0.294). A nominal association within one sex is not evidence that the slopes differ; the data therefore do not establish sex heterogeneity.

Income-group point estimates were most negative in upper-middle-income countries, near zero in lower-middle- and low-income countries, and imprecise in high-income countries. However, the joint test did not reject equality of within-country slopes across the four groups (Wald χ^2^ (3) = 4.31, *p* = 0.23). The subgroup pattern therefore does not support a statistically established income-group difference.

After correction of the country-name crosswalk, Hofstede individualism was available for 66 countries (1,412 country-years). It correlated positively with mean suicide at the country level (r = 0.26), but the fixed-effects Gini-by-individualism interaction was essentially null (b = 0.00004, *p* = 0.934). A fully decomposed hybrid model likewise found no moderation of the within-country slope (b = 0.00005, *p* = 0.912); the between-country interaction was positive but imprecise (b = 0.00089, *p* = 0.082), and the individualism main effect was imprecise (b = 0.0041, *p* = 0.397). These results did not provide convergent evidence for a stable cultural moderation pathway.

## 4 Discussion

### 4.1 Principal findings

The data contain a small negative within-country ecological signal, not evidence of absence and not evidence that inequality is protective. The primary posterior-mean and MI estimates were −0.0157 and −0.0083; their difference depends on how an external, outcome-agnostic SWIID archive is used. Population weighting preserved a similar negative point estimate. All completed-data and leave-one-country-out posterior-mean coefficients were negative, whereas temporal restrictions were smaller and imprecise, and higher-quality or reporting subsets were more negative. In light of existing cross-national studies, the central contribution is not the negative sign itself but the demonstration that its magnitude, precision, and interpretation depend materially on the estimand and analytical assumptions.

The interpretation is assumption-dependent. Country trends, region-year effects, first differences, and dynamic adjustment remove different parts of gradual national variation and have higher FMI. They may reduce confounding, remove gradual pathways, or amplify exposure noise. The observed-panel simulation shows that Rubin pooling can attenuate a true within-country slope under calibrated uncongenial scenarios, but it does not reveal the real latent Gini. We therefore present the primary pair as a sensitivity bracket and give coefficient magnitudes, intervals, and FMI priority over a threshold-based robustness label.

The calibrated conclusion is that national inequality showed a small negative within-country association whose magnitude is imprecise and dependent on exposure treatment, outcome construction, sample composition, and which temporal variation is accepted as identifying. Pooled and between-country estimates do not answer the same question. Selected high-quality subsets may reflect less attenuation, selection on development and region, or both. These are country-level patterns only. They neither show that individuals experiencing greater relative deprivation or status anxiety have higher suicide risk nor test social comparison or social cohesion as mediators, and they do not support increasing inequality.

### 4.2 Why the estimand changes the substantive interpretation

Approximately 95% of the observed variance in logged suicide lay between countries. This fact explains why a pooled coefficient cannot be read as the expected consequence of national change. It primarily compares countries with different long-run mortality environments. Welfare institutions, alcohol regulation, lethal-means availability, religious norms, urban form, health systems, migration histories, and death-registration systems all vary persistently across countries. Even an extensive covariate set cannot make those comparisons equivalent to a controlled intervention.

The within-country coefficient asks a more policy-relevant question when the claim concerns changing inequality within a country. It removes every stable national characteristic, observed or unobserved. Yet it is identified by a much smaller signal: the within-country Gini standard deviation was only 1.44 points. That small range magnifies the importance of exposure uncertainty, coding decisions, common trends, and time-varying confounding. Fixed effects improve alignment between the estimand and the substantive question, but they do not automatically convert observational macro-data into causal evidence.

This distinction has practical implications for communicating association sizes. Reporting “1.6% lower suicide per Gini point” without the alternative MI estimate, the limited within-country variation, and the uncertainty analysis invites a causal policy reading. The coefficient describes a conditional ecological association among annual national deviations. It does not describe an individual-level risk contrast or the consequence of redistribution, a tax change, or an intentional increase in inequality. Different policies can change the same Gini through different material and psychosocial pathways, none of which the present design separately identifies.

### 4.3 Interpreting the negative standard fixed-effects coefficient

Several processes could produce a negative within-country coefficient without implying a protective causal association. Inequality can co-move with growth, labor-market transformation, migration, demographic change, fiscal reform, alcohol environments, lethal-means policies, austerity, and improvements in death certification. Redistribution may also respond to crises, creating reverse timing. The four covariates capture only part of these processes. Attenuation after trends and region-year controls is compatible with slow confounding but cannot distinguish confounding from gradual substantive pathways.

A psychosocial opportunity-signal account is one possible contextual interpretation, not a mechanism tested by this analysis. During some periods of development, rising inequality may be interpreted as evidence that advancement is possible, consistent with the tunnel hypothesis; in other settings the same change may be experienced as exclusion, unfairness, or competition. The study did not observe perceived inequality, relative deprivation, mobility beliefs, social comparison, status anxiety, trust, social cohesion, belonging, or help-seeking. It therefore cannot assess mediation through these constructs or distinguish their competing interpretations. Male point estimates were more negative, but the formal sex interaction was imprecise under both exposure treatments. A mechanism should not be inferred merely because it can rationalize the sign of an ecological coefficient.

The lagged models do not establish temporal causality. One- and two-year lags remained negative, but their multiple-imputation intervals included zero. Slowly changing inequality and suicide are autocorrelated, and an annual lag does not isolate an exogenous shock. The dynamic model conditions on outcome persistence but is subject to finite-panel bias and may remove part of a genuine long-run pathway. The evidence is more consistent with a weak association embedded in gradual co-movement than with a precise short-run response.

### 4.4 Exposure and outcome measurement

SWIID’s completed series are generated from inequality sources, source relationships, welfare definitions, equivalence scales, and proximate within-country years—not from a joint model containing suicide and the present covariates. The archive is therefore uncongenial with this substantive model in the strict multiple-imputation sense (26, 27). Rubin pooling remains useful for displaying sensitivity to SWIID’s represented uncertainty, but its estimate cannot be presumed to correct exposure error or dominate the posterior-mean plug-in. Conversely, the smoothed posterior mean can overstate precision.

The estimator pattern has an analytic explanation. Regressing on a well-calibrated posterior mean induces approximately Berkson-type exposure error: the deviation of the latent value from its conditional mean is approximately independent of the mean. Hence, a linear slope is not ordinarily attenuated. Regressing on individual completed series instead introduces classical-type error around the latent exposure, which can attenuate a linear slope. This mechanism matches the simulation: the plug-in was nearly unbiased, whereas Rubin pooling attenuated the imposed coefficient by about 0.005 under both scenarios. The two bracket ends are therefore not epistemically symmetric. However, SWIID’s posterior is external and not conditioned on suicide or this covariate model, so the conditional-independence property required for pure Berkson error holds only approximately; the plug-in can still be biased or overprecise and is not treated as truth. Moreover, scenario 1 contained no plug-in exposure error, making its 0.978 coverage uninformative about exposure-error robustness. We retain −0.0157 to −0.0083 as a sensitivity bracket, not formal bounds or interchangeable estimators, alongside the wider temporal and outcome sensitivities.

Outcome construction introduces a second ambiguity. WHO directly uses usable registration for a limited set of systems and imports GBD 2021-based modeling elsewhere; GBD suicide CODEm includes alcohol, depression, development, income, education, health-care quality, and density covariates but not Gini. Larger negative estimates in the Mortality Database overlap, and narrower-interval strata can indicate less outcome and exposure error. They can also indicate selection toward high-income European reporting systems with greater variation in within-country inequality. The public MH_12 API lacks the metadata needed to classify every analytic row, so both readings remain explicit rather than resolved.

### 4.5 Public-mental-health implications

The results have methodological public-health implications before they have policy implications. National inequality is often treated as a broad contextual social determinant, but cross-country correlations and within-country change coefficients are different forms of evidence, and neither directly tests individual psychosocial mechanisms. Reviews, dashboards, and policy briefs should state which variation underpins their conclusions. When most outcome variance is between countries and the exposure is slow-moving, analyses should specify within-country variation, compare within- and between-estimands, and assess sensitivity to treatment of exposure-uncertainty, outcome measurement, sample composition, and temporal assumptions. This sensitivity of interpretation—rather than a new claim about the direction of the association—is the study’s principal contribution.

For suicide prevention, the findings provide no rationale for increasing inequality. The negative standard TWFE coefficient is too model-dependent to support such an intervention, and a Gini coefficient is not a manipulable treatment. Redistribution, social insurance, labor-market policy, minimum wages, taxation, and public services alter multiple material and psychosocial pathways simultaneously. Their mental-health effects must be evaluated at the policy level rather than inferred from the sign of an ecological Gini coefficient. Communication of these findings should therefore avoid translating a country-level coefficient into claims that inequality is psychologically beneficial or protective at the individual level.

The analysis also underscores the value of surveillance infrastructure. Countries with weak mortality registration produce modeled outcomes with wider uncertainty, and inequality estimates depend on survey coverage and harmonization. Investments in civil registration, cause-of-death certification, household income measurement, and transparent reporting of uncertainty improve both national prevention and comparative research. Precision restrictions cannot substitute for such investments because they selectively exclude countries with the greatest data limitations and narrow the global relevance of the analysis.

The sex analysis shows that moderators require a model that permits the full adjustment structure and time effects to vary across groups, followed by the same exposure-uncertainty treatment used for the main association. Male point estimates were more negative, but the formal difference remained imprecise at the posterior mean and across the SWIID completed datasets. This does not prove that male and female responses to labor-market conditions or status processes are identical; it shows that these data cannot establish different inequality slopes. More informative public-mental-health studies should connect policy and macroeconomic change to age-, sex-, and occupation-specific mortality and to proximal psychosocial measures.

### 4.6 Strengths and limitations

This study has several strengths. It covers 170 countries over up to 22 years and retains 87% of the potential balanced panel. It distinguishes pooled, between-country, and within-country estimands, reports staged adjustments, and tests several temporal-identification assumptions. The complete SWIID version 9.92 archive was matched exactly to the analytic panel and carried through principal estimands, sample restrictions, lags, market-income inequality, outcome validation, and the sex interaction. The study also quantifies identifying variation, examines influential countries and regions, and provides executable, machine-readable reproduction materials.

The limitations are substantial. This ecological design cannot translate national coefficients into individual suicide risk, infer individual-level relationships from country-level associations, or test whether relative deprivation, status anxiety, social comparison, social cohesion, or related psychosocial constructs mediate the observed pattern. These theories offer only possible contextual interpretations. Fixed effects do not eliminate time-varying confounding from alcohol environments, social expenditure, tax and transfer reform, health-system change, migration, conflict, lethal-means regulation, certification, or age-specific labor markets. Adding World Bank alcohol consumption to the available 3,081 rows changed the MI coefficient only from −0.00848 to −0.00821, but this does not settle alcohol’s causal role, and the indicator may overlap with outcome-model predictors.

Causal ordering remains uncertain. Inequality, unemployment, fiscal policy, and suicide can jointly respond to shocks; lagged exposure is not an instrument. SWIID is harmonized and partly model-based, and its externally completed series are not congenial with the present outcome model. Rubin pooling propagates uncertainty represented by SWIID but may attenuate a substantive slope under some data-generating mechanisms; the posterior mean may instead be too smooth. Neither approach corrects unrepresented bias.

Suicide outcomes remain imperfect. WHO and GBD share registration inputs and modeling, whereas reported Mortality Database rates are affected by underregistration and classification. The MH_12 public extract lacks row-level input-source flags, so the exact number of analytic country-years based mainly on direct versus modeled inputs is unavailable. The Mortality Database overlap is transparent but selected and broader than WHO’s 66 system-usable-registration set. The unbalanced complete-case panel also contracts global representation, especially in later years.

The study was not supported by a preregistration or a dated prospective analysis plan in the archived materials. The fully adjusted unweighted TWFE model is now explicitly primary; population weighting, reporting decompositions, alcohol adjustment, equivalence testing, and simulation were added at revision and are labeled as sensitivity or exploratory analyses. No multiplicity correction was applied. Accordingly, subgroup *p*-values and the male slope are not treated as independent confirmatory findings; the formal male–female interaction remained imprecise. The exact minimum detectable coefficients were 0.01223 at 80% power and 0.01415 at 90% power, both larger than the *post hoc* SESOI of 0.010. The study therefore could not reliably detect an association at the magnitude it declared minimally interesting, and the TOST was not positioned to establish equivalence within that margin. This limitation is more informative about the study’s imprecision than whether any one *p*-value crosses 0.05.

Finally, each sensitivity specification targets a different estimand. Country trends, region-year effects, first differences, and dynamic adjustment can remove both confounding and meaningful causal variation. The loss of precision under those models does not prove the posterior-mean TWFE estimate is spurious. It shows that the substantive conclusion depends on whether gradual co-movement is treated as signal or nuisance. That dependence is itself a central result.

### 4.7 Directions for future research

Stronger evidence requires designs that link macro-distributional change to individual and institutional pathways. Repeated cross-national surveys could measure perceived inequality, reference groups, mobility expectations, status anxiety, trust, belonging, financial strain, mental disorders, suicidal ideation, and help-seeking within the same countries and years. Multilevel models could then test whether objective inequality predicts these proximal variables and whether these associations vary by income position, age, sex, occupation, or welfare regime. These designs could also combine country-year inequality and digital-connectivity indicators with individual measures of media use and message exposure. Aggregate search or platform data could provide complementary contextual evidence, but should not substitute for individual-level measurement.

Policy-based quasi-experiments offer another avenue. Tax reforms, transfer expansions, minimum-wage changes, pension reforms, labor-market protections, and social-insurance shocks can yield more interpretable variation than annual Gini movements. Event-study and synthetic-control designs should assess pre-trends, policy anticipation, concurrent reforms, and differential exposure. Because many policies affect both the income distribution and material security, researchers should specify whether they seek the total policy effect, the portion mediated by inequality, or the effect of distributional change while holding other pathways constant.

Where possible, improved mortality data should be linked to age- and sex-specific measures of inequality or income distributions. Subnational panels can increase temporal and geographic variation, but they also require careful attention to migration, spillovers, and local reporting. Distributional measures beyond the Gini—including lower-tail deprivation, top shares, polarization, and inequality of opportunity—may align more closely with specific mechanisms. Qualitative and experimental studies are necessary to determine whether objective changes are perceived as exclusion, unfairness, competition, or opportunity.

Future analyses should also treat measurement uncertainty as part of the substantive problem. When an exposure database provides posterior draws, downstream models should carry those draws through rather than using only a summary mean. Repositories should include source versions, crosswalks, exclusion logs, and scripts that reproduce both the analytic panel and the reported tables. These practices make disagreements about modeling assumptions visible and allow alternative estimands to be evaluated without rebuilding the entire dataset.

## 5 Conclusion

Across 170 countries from 2000 to 2021, the data support a small negative within-country ecological association between disposable-income inequality and suicide mortality, but the size is imprecise and assumption-dependent. The primary posterior-mean and SWIID MI estimates bracketed b = −0.0157 to −0.0083; population weighting preserved the negative point estimate, whereas trajectory-adjusted and short-run models yielded smaller and less precise estimates. Higher-quality and reporting subsets were more negative but were strongly selected on income, region, and statistical capacity. The study does not test individual mechanisms such as relative deprivation, status anxiety, social comparison, or social cohesion; these remain possible contextual interpretations that would require individual-level or multilevel data. The principal contribution is to show that the meaning of the country-level association changes with the estimand, exposure-uncertainty treatment, outcome measurement, sample composition, and temporal identifying assumptions.

## Data Availability

The datasets presented in this study can be found in online repositories. The names of the repository/repositories and accession number(s) can be found: https://doi.org/10.5281/zenodo.21848348.
