# Supplementary material for: National income inequality and suicide mortality across 170 countries, 2000–2021: within-country evidence and sensitivity to exposure uncertainty, with implications for health communication
Source: Front Public Health. 2026 Sep 8;14:1915415. doi: 10.3389/fpubh.2026.1915415 (PMC13593783; doi:10.3389/fpubh.2026.1915415)
Supplement: Supplementary file 1 [file Supplementary_file_1.docx]

**Supplementary Material**

Scope and methods

WHO age-standardized suicide rates were linked to SWIID version 9.92 disposable-income Gini estimates for 170 countries from 2000–2021. The primary specification was an unweighted, fully adjusted two-way fixed-effects model with country and year indicators and country-clustered standard errors. Exposure uncertainty was propagated across all 100 completed SWIID series using Rubin’s rules and Barnard–Rubin degrees of freedom. The design is ecological: the estimates describe within-country population associations and do not identify individual-level mechanisms or causal effects.

**Supplementary Table S1. Coverage and primary exposure-uncertainty results.**

| **Item** | **Value** |
| --- | --- |
| Study period | 2000–2021 |
| Countries | 170 |
| Country-years | 3,257 |
| Observed share of the potential panel | 87.1% |
| Countries observed in all 22 years | 93 |
| Within-country SD of disposable Gini (posterior mean) | 1.440 |
| Primary posterior-mean TWFE | −0.0157 (95% CI [−0.029, −0.003]; p = .018) |
| Primary SWIID multiple-imputation TWFE | −0.00831 (95% CI [−0.01688, 0.00026]; p = 0.057) |
| Fraction of missing information | 0.172 |
| Completed-data coefficient range | −0.01296 to −0.00385 |

*Note. TWFE, two-way fixed effects; CI, confidence interval. The completed-data range summarizes the 100 SWIID completed series and is not a confidence interval.*

**Supplementary Table S2. Key sensitivity estimates after SWIID uncertainty propagation.**

| **Check** | **Specification** | **b** | **95% CI** | **p** | **Country-years** |
| --- | --- | --- | --- | --- | --- |
| Primary | Fully adjusted TWFE | −0.0083 | [−0.0169, 0.0003] | 0.057 | 3,257 |
| Temporal identification | Country-specific linear trends | −0.0025 | [−0.0104, 0.0054] | 0.529 | 3,257 |
| Temporal identification | Country FE + WHO region-year FE | −0.0042 | [−0.0126, 0.0042] | 0.325 | 3,257 |
| Temporal identification | First differences | −0.0003 | [−0.0051, 0.0044] | 0.892 | 3,075 |
| Temporal identification | Dynamic FE + lagged outcome | −0.0016 | [−0.0042, 0.0010] | 0.224 | 3,075 |
| Panel composition | Balanced panel | −0.0115 | [−0.0231, 0.0001] | 0.052 | 2,046 |
| Period | Period ends in 2019 | −0.0088 | [−0.0176, 0.0000] | 0.051 | 3,034 |
| Outcome precision | Narrowest half of WHO intervals | −0.0150 | [−0.0283, −0.0017] | 0.028 | 1,725 |
| Exposure precision | Narrowest half of SWIID SEs | −0.0140 | [−0.0274, −0.0006] | 0.041 | 1,810 |
| Lag | Gini lagged 1 year | −0.0081 | [−0.0167, 0.0004] | 0.063 | 3,075 |
| Outcome | GBD/IHME, same analytic sample | −0.0062 | [−0.0132, 0.0007] | 0.079 | 3,257 |
| Outcome | WHO Mortality Database, reporting subset | −0.0218 | [−0.0467, 0.0030] | 0.084 | 1,739 |

*Note. Every row pools all 100 SWIID completed datasets. Precision-restricted rows are selected-sample checks and should not be interpreted as unbiased quality corrections. FE, fixed effects; GBD, Global Burden of Disease; SE, standard error.*
